# Agglomeration of titanium dioxide nanoparticles increases toxicological responses in vitro and in vivo

**DOI:** 10.1186/s12989-020-00341-7

**Published:** 2020-02-26

**Authors:** Sivakumar Murugadoss, Frederic Brassinne, Noham Sebaihi, Jasmine Petry, Stevan M. Cokic, Kirsten L. Van Landuyt, Lode Godderis, Jan Mast, Dominique Lison, Peter H. Hoet, Sybille van den Brule

**Affiliations:** 1 Laboratory of Toxicology, Unit of Environment and Health, Department of Public Health and Primary Care, KU Leuven, 3000 Leuven, Belgium; 2 Trace Elements and Nanomaterials, Sciensano, 1180 Uccle, Belgium; 3 National Standards, FPS Economy, 1000 Brussels, Belgium; 4 Department of Oral Health Sciences, KU Leuven, BIOMAT & UZ Leuven (University Hospitals Leuven), Dentistry, Kapucijnenvoer 7, 3000 Leuven, Belgium; 5 Laboratory for Occupational and Environmental Hygiene, Unit of Environment and Health, Department of Public Health and Primary Care, KU Leuven, 3000 Leuven, Belgium; 6 IDEWE, External Service for Prevention and Protection at work, Interleuvenlaan 58, 3001 Heverlee, Belgium; 7 Louvain centre for Toxicology and Applied Pharmacology, Institute of Experimental and Clinical Research, Université catholique de Louvain, 1200 Brussels, Belgium

**Keywords:** Nanomaterials, Titanium dioxide, Agglomerates, Toxicity, Biological responses

## Abstract

**Background:**

The terms agglomerates and aggregates are frequently used in the regulatory definition(s) of nanomaterials (NMs) and hence attract attention in view of their potential influence on health effects. However, the influence of nanoparticle (NP) agglomeration and aggregation on toxicity is poorly understood although it is strongly believed that smaller the size of the NPs greater the toxicity. A toxicologically relevant definition of NMs is therefore not yet available, which affects not only the risk assessment process but also hinders the regulation of nano-products. In this study, we assessed the influence of NP agglomeration on their toxicity/biological responses in vitro and in vivo.

**Results:**

We tested two TiO_2_ NPs with different primary sizes (17 and 117 nm) and prepared ad-hoc suspensions composed of small or large agglomerates with similar dispersion medium composition. For in vitro testing, human bronchial epithelial (HBE), colon epithelial (Caco2) and monocytic (THP-1) cell lines were exposed to these suspensions for 24 h and endpoints such as cytotoxicity, total glutathione, epithelial barrier integrity, inflammatory mediators and DNA damage were measured. Large agglomerates of 17 nm TiO_2_ induced stronger responses than small agglomerates for glutathione depletion, IL-8 and IL-1β increase, and DNA damage in THP-1, while no effect of agglomeration was observed with 117 nm TiO_2_.

In vivo, C57BL/6JRj mice were exposed via oropharyngeal aspiration or oral gavage to TiO_2_ suspensions and, after 3 days, biological parameters including cytotoxicity, inflammatory cell recruitment, DNA damage and biopersistence were measured. Mainly, we observed that large agglomerates of 117 nm TiO_2_ induced higher pulmonary responses in aspirated mice and blood DNA damage in gavaged mice compared to small agglomerates.

**Conclusion:**

Agglomeration of TiO_2_ NPs influences their toxicity/biological responses and, large agglomerates do not appear less active than small agglomerates. This study provides a deeper insight on the toxicological relevance of NP agglomerates and contributes to the establishment of a toxicologically relevant definition for NMs.

## Background

Manufactured nanomaterials (NMs) exist as unbound (single) particles, agglomerates, aggregates or as a mixture thereof [1–4]. This is clearly recognised in the definition of NMs recommended by the European Union (EU) stating “*manufactured material containing particles, in an****unbound state or as an aggregate or as an agglomerate****and where, for 50 % or more of the particles in the number size distribution, one or more external dimensions is in the size range****1 nm-100 nm***” [5]. This definition was proposed for legislative and regulatory purposes with no direct regard to hazard. Although agglomerates and aggregates (AA) are often erroneously considered similar and interchangeably used, they are, however, two different secondary structures of particulate materials. In agglomerates, the particles bind together by weak forces, which are reversible, while, in aggregates, particles fuse irreversibly together [6]. The terms AA attracted in recent years attention among the NM producers, consumers, regulatory authorities and policy makers in view of their potential influence on human health effects [5, 7, 8]. However, no sound scientific data justify that AA may or may not be relevant from a toxicological perspective. This knowledge gap is not only affecting the risk assessment process but also hindering the development of guidelines to regulate NMs in commercial products.

Agglomeration in particular, is a ubiquitous phenomenon and its dynamic behaviour poses a great challenge in assessing health impacts [9, 10]. Unlike aggregates, agglomerates are very sensitive to changes in the environment such as pH, ionic strength, presence of proteins and motion of the carrier medium, and can de-agglomerate/agglomerate further depending on the environment [10, 11]. While this induces complex behaviour of NMs in exposure scenarios and in tissue uptake and bio-distribution, influence on toxicity/biological responses remain poorly understood [9, 10].

Titanium dioxide (TiO_2_) is one of the most abundantly produced NMs and is used in food, paints and in personal care products [12, 13]. Humans are increasingly exposed to TiO_2_ via inhalation, dermal or oral exposure. Based on animal studies, the International Agency for Research on Cancer (IARC) classified TiO_2_ as a group 2B carcinogen (possibly carcinogenic to humans) [14]. Very recently, the French agency for food, environmental and occupational health and safety (ANSES) banned the use of TiO_2_ as a food additive (E171) due to its genotoxic potential [15]. While several studies showed that TiO_2_ NPs can induce adverse effects including DNA damage and chromosomal damage, findings are contradictory [16, 17]. TiO_2_ NPs are well known for their agglomeration and, so far, extensive efforts have been dedicated at minimizing agglomeration using different dispersion protocols to assess their toxicity despite a lack of evidence that agglomeration influences their toxicity/biological responses.

In this study, we aimed to determine the influence of agglomeration state of TiO_2_ NPs on toxicity/biological effects. Toxicological studies generally suggest that the smaller the size of the primary NPs the greater the toxicity/biological responses [18–21]. ***Therefore*,*we hypothesized that smaller agglomerates of NPs induce stronger toxicity/biological responses compared to their largely agglomerated counterparts.*** To test this hypothesis, we selected two TiO_2_ NPs of identical phase, coating and chemical composition but with different primary particle size and compared their toxicity in different agglomeration states using in vitro *and* in vivo models.

## Results

### Dispersions and size characterization of TiO_2_ NP agglomerate suspensions

**Fig. 1:**
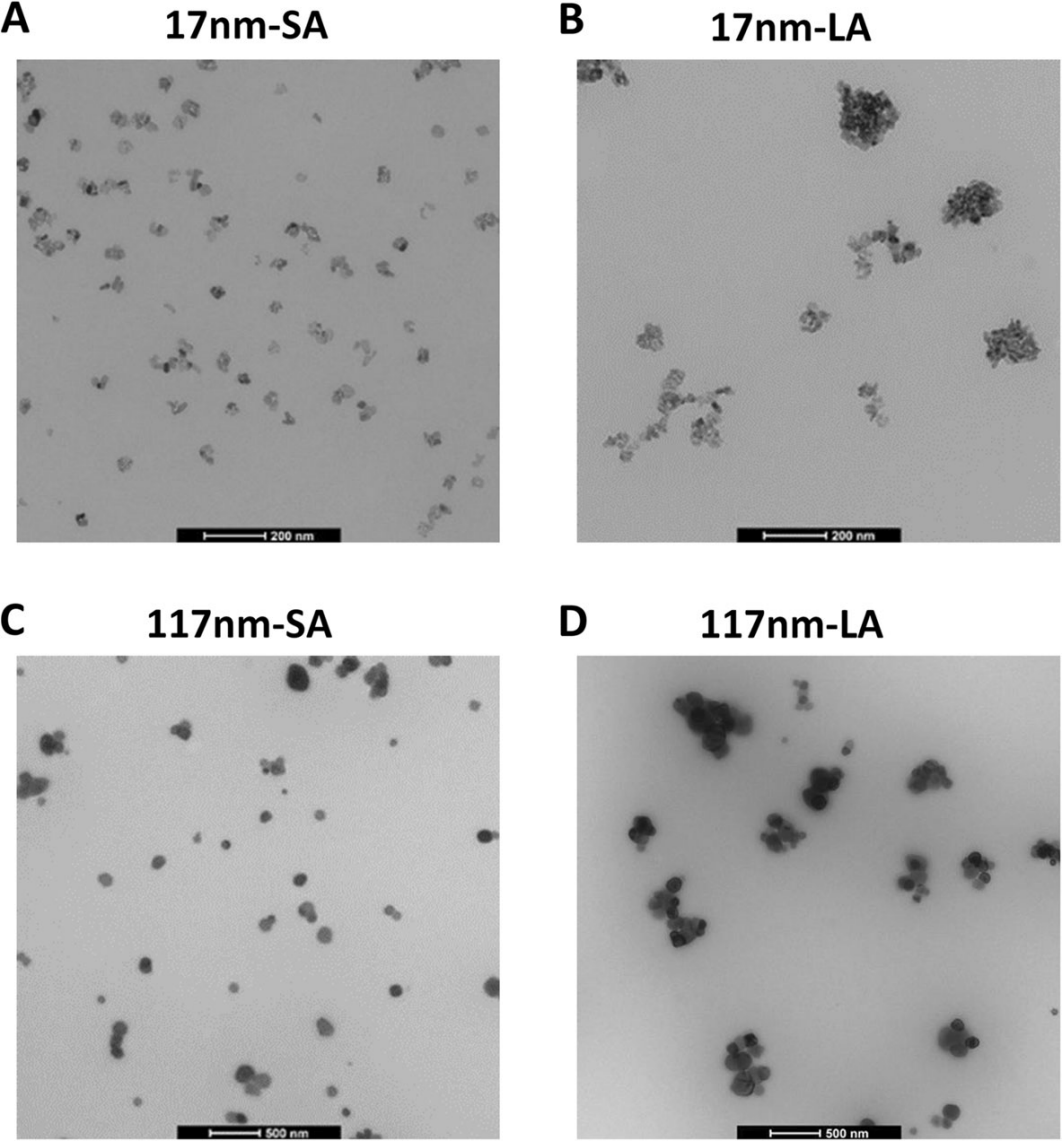
Representative TEM micrographs of freshly prepared TiO_2_ stock suspensions of small (SA) and large agglomerates (LA). 17 nm-SA (**a**), 17 nm-LA (**b**), 117 nm-SA (**c**) and 117 nm-LA (**d**)

Our strategy to prepare ad-hoc stable suspensions of TiO_2_ NPs with different agglomeration states, in the same dispersion medium, was based on the method developed by Guiot and Spalla [22] (illustrated in Additional file 1: Figure S2). Figure 1 shows representative Transmission Electron Microsopy (TEM) micrographs of the freshly prepared TiO_2_ stock suspensions. The 17 nm sized TiO_2_ at pH 2 was relatively well dispersed and predominantly existed as small aggregates **(indicated as 17 nm-SA)** compared to the suspension prepared at pH 7.5, in which particles tend to agglomerate strongly **(17 nm-LA)**. In contrast, 117 nm TiO_2_ were found to be less agglomerated when dispersed at pH 7.5 **(117 nm-SA)** and existed as large agglomerates when dispersed at pH 2 solution **(117 nm-LA)**. After dispersion in the respective pH conditions, TiO_2_ suspensions were sonicated at constant energy (7056 J) and stabilized immediately using bovine serum albumin (BSA, 0.25%). The suspensions dispersed at pH 2 were readjusted to pH 7–7.5 before size characterization and cell/animal exposure.

Sizes of TiO_2_ suspensions are presented in Table 1. TEM analyses showed that the median Equivalent Circle Diameter (ECD) of 17 nm-SA and 117 nm-SA were 18 and 122 nm, respectively. The TiO_2_ NPs were, thus, in their most dispersed state in these suspensions. Median ECD of the large agglomerates, 17 nm-LA and 117 nm-LA, were 127 and 352 nm respectively, clearly indicating that NPs were more agglomerated in these suspensions. Mean ECD were substantially different: 100 nm for 17 nm-SA; 200 nm for 117 nm-SA; 250 nm for 17 nm-LA; and 500 nm for 117 nm-LA, confirming the overestimation of sizes when means are used. TEM was also applied to measure mean Feret minimum (Feret min) and the measured sizes were slightly different compared to median ECD (Table 1). The mean hydrodynamic diameter (Z-average) measured by dynamic light scattering (DLS) showed larger sizes for 17 nm TiO_2_ (SA 600 nm; LA 900 nm) than 117 nm stock suspensions (SA 280 nm; LA 580 nm). Hydrodynamic sizes measured using particle tracking analysis (PTA) were smaller than Z-average in sizes for 17 nm TiO_2_ suspensions (SA 134 and LA 207 nm) and 117 nm TiO_2_ suspensions (SA 259 and LA 221 nm).

**Table 1:** Size characterization of freshly prepared TiO_2_ stock suspensions (2.56 mg/mL)

Stock suspensions	TEM			DLS	PTA
	Median ECD (nm)	Mean ECD (nm)	Mean Feret min (nm) ± SD	Z-average (nm)	Mean hydrodynamic size (nm)
17 nm-SA	18	100	33 ± 2	600	134
17 nm-LA	127	200	120 ± 19	900	207
117 nm-SA	122	250	148 ± 10	280	259
117 nm-LA	352	500	309 ± 64	580	221

Median and mean equivalent circle diameter (ECD) and mean feret minimum (feret min) measured by transmission electron microscopy (TEM), Z-average (mean hydrodynamic size) by dynamic light scattering (DLS) and mean hydrodynamic size by particle tracking analysis (PTA)

*SD* standard deviation

The stability of these suspensions in exposure media was measured by DLS (Table 2). After dilution to 100 μg/mL, Z-averages were measured directly and after 24 h. In DMEM/F12 (typically used for HBE cell cultures) and RPMI 1640 (used for THP-1) only a slight change was observed after 24 h of incubation. In DMEM/HG (used for Caco2), at least a two-fold increase of Z-average after 24 h incubation was noted. The polydispersity index (PDI) was less than ~ 0.35 in stock suspensions and in cell culture medium at 0 and 24 h, indicating an acceptable distribution of sizes and good stability of these suspensions.

**Table 2:** Size characterization of TiO_2_ in stock and exposure media (HBE,Caco2 and THP-1) using DLS

		Stock		DMEM/F12 (HBE)		DMEM/HG (Caco2)		RPMI 1640 (THP-1)	
		Z-avg	PDI	Z-avg	PDI	Z-avg	PDI	Z-avg	PDI
17 nm-SA	0 h	600	0.34	670	0.27	630	0.31	1140	0.18
	24 h	600	0.35	850	0.24	1580	0.28	1035	0.22
17 nm-LA	0 h	900	0.42	900	0.27	870	0.30	1350	0.30
	24 h	800	0.40	980	0.20	1546	0.24	1330	0.25
117 nm-SA	0 h	280	0.18	690	0.19	547	0.18	1010	0.18
	24 h	290	0.19	750	0.20	1145	0.40	900	0.18
117 nm-LA	0 h	580	0.36	630	0.26	630	0.26	880	0.30
	24 h	590	0.37	650	0.21	1300	0.57	960	0.23

Stock suspensions (2.56 mg/mL) were diluted to 100 μg/mL in different cell culture medium and, hydrodynamic sizes (Z-avg) and poly dispersity index (PDI) were measured directly and after 24 h

In conclusion, TEM indicated a clear difference between SA and LA for both TiO_2_ NPs and stock suspensions were found to be stable over 24 h using DLS, indicating that these ad-hoc suspensions were appropriate to test our hypothesis.

### Influence of TiO_2_ agglomeration on in vitro dosimetry

**Fig. 2:**
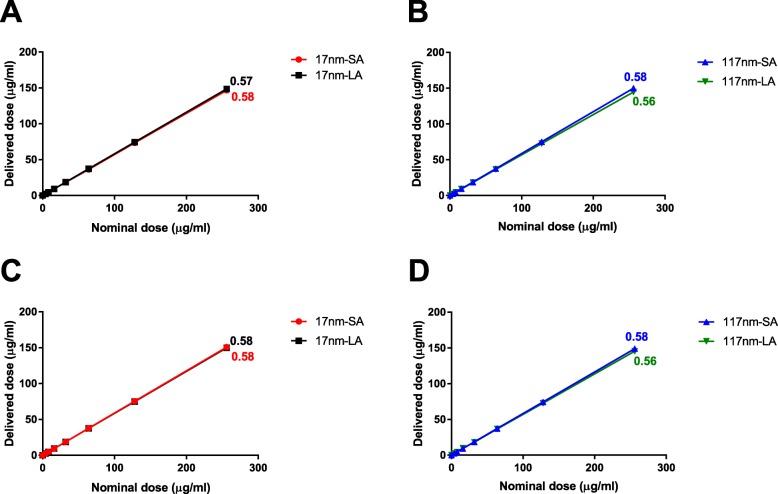
Estimated TiO_2_ dose reaching the bottom of the wells after 24 h as a function of increasing nominal doses applied in exposure media. Dosimetry simulation was performed with a distorted grid (DG) model for 17 (**a** and **c**) and 117 nm (**b** and **d**) using parameters obtained from exposure media DMEM/F12 (**a** and **b**) and RPMI 1640 (**c** and **d**). The slope values are indicated near the respective lines. *R*^*2*^ > 0.99 for all the suspensions. The percentage of dose delivered to the cells did not differ for 96 and 24 well plates, as the height of the liquid column was similar (6 mm)

Before examining biological responses to these differently agglomerated suspensions, we considered the possible influence of differential sedimentation of the suspensions in vitro, which might confound the cell responses. In vitro dosimetry simulation was performed only using DMEM/F12 (used for HBE) and RPMI 1640 (used for THP-1) because DMEM/HG (used for Caco2) promoted further agglomeration over 24 h incubation. The main parameters used to perform dosimetry simulation are listed in Additional file 1: Table S1. Figure 2 shows the estimated TiO_2_ dose reaching the bottom of the wells as a function of nominal (applied) dose. Regardless of the type of exposure medium and TiO_2_ primary size/agglomeration state, nearly 56–58% of the applied doses was delivered to the bottom of the wells after 24 h. Thus, the delivered doses between SA and their LA suspensions of both TiO_2_ did not differ substantially. The results are, therefore, presented as a function of nominal doses (expressed in μg/mL).

### Comparison of biological responses

Since inhalation and ingestion are the primary routes of exposure to these NPs during production and use, we studied the in vitro effects in human bronchial (HBE) and colon (Caco2) epithelial cell lines, respectively. In addition, we used a human monocytic cell line (THP-1) as a representative of innate immune cells that are actively involved in phagocytosis of these particles. To investigate the acute toxicity in vivo, oropharyngeal and gavage administrations were used as representative for inhalation and ingestion, respectively.

In order to investigate the validity of our hypothesis, we first determined the endpoints for which responses to TiO_2_ exposure (for both SA and LA suspensions) were statistically different compared to untreated control using one-way ANOVA. Table 3A and B summarise these results in vitro and in vivo*,* respectively. If no impact of TiO_2_ treatment on a given endpoint in both agglomeration states (SA and LA) was revealed, such endpoint was not used to test the hypothesis of the influence of agglomerations. In a second step, we analysed only those endpoints where TiO_2_ induced a significant effect at least in one of the agglomeration states (SA or LA). We compared the effects induced by SA and LA using two-way ANOVA. If differences were observed between suspensions, a post hoc test (Bonferroni’s multiple comparison test) was used to determine the suspension that induces the strongest effect at the same mass concentration/dose (see Table 4A and B).

**Table 3:** Summary of the in vitro (A) and in vivo (B) responses to TiO_2_ exposure

(A)						
In vitro responses to TiO_2_ exposure						
Biological endpoint	HBE		Caco2		THP-1	
	17 nm	117 nm	17 nm	117 nm	17 nm	117 nm
Cell metabolic activity	No	No	No	No	No	No
Cell viability	No	No	No	No	No	No
DNA damage	Yes	Yes	Yes	Yes	Yes	Yes
GSH	Yes	Yes	Yes	No	Yes	No
TEER	Yes	Yes	No	Yes	n/a	n/a
IL-8	No	No	No	No	Yes	No
IL-6	Yes	No	No	No	No	No
TNF-α	No	No	No	No	Yes	No
IL-1β	No	Yes	No	No	Yes	No
(B)						
In vivo responses to TiO_2_ exposure						
Biological endpoint	Aspiration		Gavage			
	17 nm	117 nm	17 nm	117 nm		
BAL cell number	No	No	n/a	n/a		
BALF LDH	No	Yes	n/a	n/a		
BALF proteins	No	No	n/a	n/a		
BAL macrophages	No	No	n/a	n/a		
BAL neutrophils	No	No	n/a	n/a		
BAL lymphocytes	Yes	Yes	n/a	n/a		
Blood lymphocytes	No	No	No	No		
Blood monocytes	No	No	No	No		
Blood granulocytes	No	No	No	No		
Lung Ti	Yes	Yes	n/a	n/a		
Blood Ti	No	No	No	No		
BAL DNA damage	No	No	n/a	n/a		
Blood DNA damage	n/a	n/a	Yes	Yes		
GSH lung	No	No	n/a	n/a		
GSH liver	n/a	n/a	No	No		

“Yes” indicates *p* < 0.05 (One-way ANOVA) and a significant difference compared to control; “No” indicates *p* > 0.05; n/a-not available

**Table 4:**
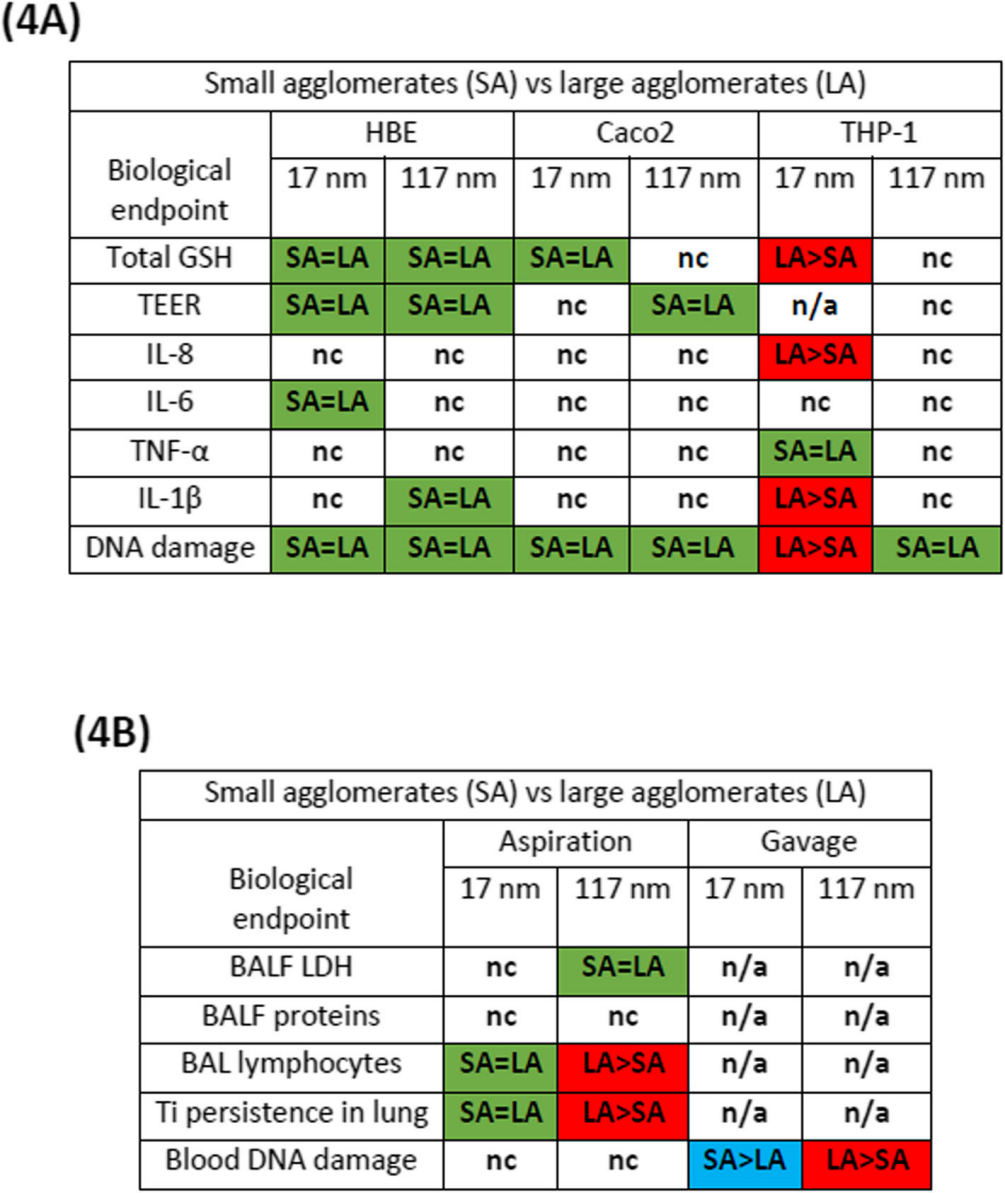
Summary of in vitro (A) and in vivo (B) responses to differently agglomerated TiO_2_ suspensions

SA = LA (indicated in green) when *p* > 0.05; LA > SA (red) or SA > LA (blue) when *p* < 0.05(Two-way ANOVA). When suspensions are statistically different, a post hoc - Bonferroni’s multiple comparison test was used to statistically determine whether LA or SA induced a stronger effect at the same mass concentrations/doses; nc- not compared as both suspensions did not induce any significant activity compared to control. n/a-not available

**Fig. 3:**
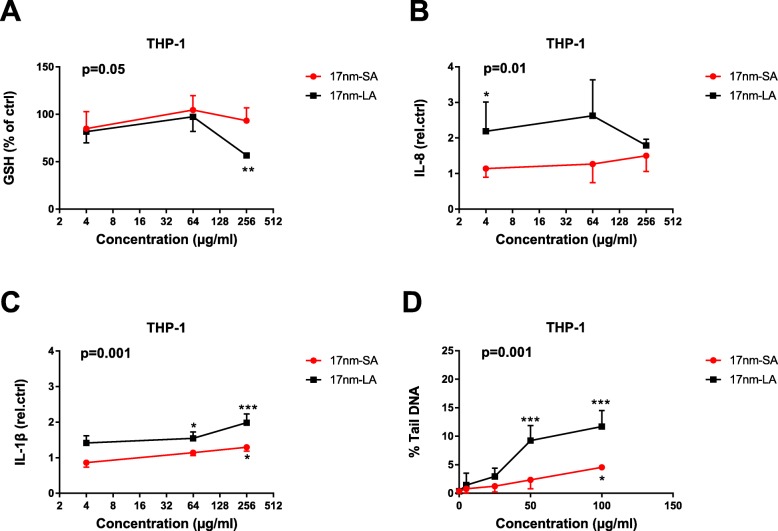
Influence of TiO_2_ agglomeration on THP-1 biological responses. Total glutathione (GSH) (**a**), IL-8 (**b**) and IL-1β secretion (**c**), and DNA damage (**d**) measured in cell cultures after 24 h exposure to different concentrations of small (SA) and large agglomerates (LA) of 17 nm TiO_2_. Data are expressed as means ± SD from three independent experiments performed in duplicates. *p* < 0.05 (*), *p* < 0.01 (**) and *p* < 0.001 (***) represent significant difference compared to control (One-way ANOVA followed by Dunnett’s multiple comparison test). Two-way ANOVA was used to determine the significant differences between suspensions (significant p value indicated at the top left corner)

**Fig. 4:**
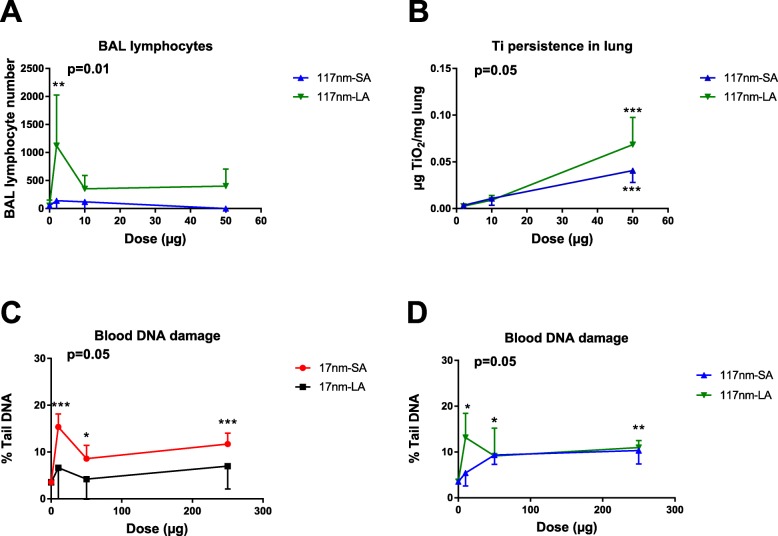
Influence of TiO_2_ agglomeration on in vivo responses in mice exposed via oropharyngeal aspiration or oral gavage. BAL lymphocytes (**a**) and Ti persistence in lung tissues (**b**) in aspirated mice and blood DNA damage in gavaged mice (**c** and **d**) measured 3 d after exposure to increasing doses of small (SA) and large agglomerates (LA) of 17 and 117 nm TiO_2_. Data are expressed as means ± SD from 4 to 5 mice in each group. *p* < 0.05 (*), *p* < 0.01 (**) and *p* < 0.001 (***) represent significant difference compared to control (One way ANOVA followed by Dunnett’s multiple comparison test). Two-way ANOVA was used to determine the significant differences between suspensions (significant *p* value indicated at the top left corner)

The results indicated in green (SA = LA) are shown in Additional file 1: Figures S3 to S8. Significant results indicated in red or blue in Table 4, are presented in Fig. 3 for the in vitro experiments (total glutathione, IL-8, IL-1β and DNA damage in THP-1 exposed to 17 nm TiO_2_) and in Fig. 4 for the in vivo experiments (lymphocytes in the broncho-alveolar lavage and Ti persistence in lung tissue for aspirated mice with 117 nm TiO_2_, Fig. 4a and b; blood DNA damage in gavaged mice for 17 and 117 nm TiO_2_, Fig. 4c and d).

In HBE and Caco2 cells, the biological effects, including DNA damage, of SA and LA for both TiO_2_ samples did not vary (see Additional file 1: Figures S3-S7). In contrast, in THP-1, LA of 17 nm TiO_2_ induced stronger GSH depletion, secretion of IL-8 and IL-1β and more DNA strand breaks compared to SA (Fig. 3). Such differences between SA and LA were not observed for the 117 nm TiO_2_ NPs in THP-1 cells. In vivo, LA of 117 nm TiO_2_ induced a significantly stronger increase of BAL lymphocytes than SA (Fig. 4a). Further, Ti detected in the lung after 3 d was significantly higher for LA of 117 nm TiO_2_ (Fig. 4b). Such differences between SA and LA were not noticed in mice exposed with 17 nm TiO_2_ (see Additional file 1: Figure S8A and S8B). In gavaged mice, SA of 17 nm TiO_2_ and LA of 117 nm TiO_2_ induced significantly higher blood DNA damage compared to their counterparts (Fig. 4c and d).

## Discussion

The focus of this study was to compare the magnitude of the toxicity/biological responses induced by small (SA) and large agglomerates (LA) of two TiO_2_ NPs with different primary particle sizes. In in vitro testing, differential responses were observed only in THP-1 cells, where LA of 17 nm TiO_2_ induced stronger biological responses than SA. In in vivo testing*,* LA of 117 nm TiO_2_ induced stronger pulmonary effects and blood DNA damage compared to the SA. These results contradict our initial hypothesis as small TiO_2_ agglomerates did not necessarily appear more toxic/biologically active than their large counterparts.

To systematically determine the influence of NP agglomeration on toxicity, the thorny task was to develop standardized protocols to reproducibly generate agglomeration. Most importantly, this had to be done with minimal/negligible variation in the dispersion media, to avoid bias due to medium effects. In the past, some attempts were made to determine the influence of TiO_2_ NP agglomeration on toxicity using different protocols. Magdolenova et al. 2012 [23] prepared two suspensions; a well-dispersed condition of TiO_2_ NPs in the presence of serum proteins (20% FBS) and an unstable/agglomerated condition without serum proteins and found that the large agglomerates induced DNA damage in three different cell lines while the small agglomerates did not. Prasad et al. 2013 [24] investigated the effect of three different culture media (with variable amount of BSA and FBS) on agglomeration and observed that TiO_2_ induced micronuclei in its least agglomerated condition. Lankoff et al. 2012 [25] also used BSA (15%) and FBS (10%) and obtained two suspensions with differently agglomerated state, but, in contrast to the two above mentioned studies, these authors did not observe any differences in A549 cell death between the two suspensions. Thus, within the same study, different media and protein concentrations were used to produce suspensions with different states of agglomeration, which is an evident source of potential bias [26] and probably, influenced the toxicological outcome. Therefore, in our study, we decided to vary only the pH of the initial dispersion medium to modify the agglomeration state of the TiO_2_ NPs. After stabilization of these dispersions with BSA, the pH was readjusted to obtain a pH compatible with toxicological tests. At the same pH, 17 and 117 nm TiO_2_ existed in different agglomeration state because the isoelectric point of TiO_2_ NPs varies according to primary size [27]. Thus, we succeeded to produce TiO_2_ NPs in different agglomeration states suitable for testing our hypothesis without experimental bias. Most importantly, we used exactly the same amount of sonication energy (7056 J) and proteins (final BSA concentration, 0.25%) to disperse and sterically stabilize the differently agglomerated suspensions. To the best of our knowledge, this is the first attempt to produce different agglomeration states of TiO_2_ NPs with minimal changes in dispersion media, and thus a more reliable approach to study the influence of agglomeration on toxicity.

To characterize the cytotoxic activity of these suspensions in vitro, we investigated the effect on the cell metabolic activity and viability after 24 h exposure. Both TiO_2_ NPs were not cytotoxic even in their most dispersed state. Then we compared the potential of SA and LA to induce an oxidative stress, pro-inflammatory responses and DNA damage, or to disturb the epithelial barrier integrity. The agglomeration state of both TiO_2_ did not influence these responses in HBE and Caco2 cell cultures. In contrast to these cell lines, a distinct response was noticed in THP-1 cells, where LA of 17 nm TiO_2_ were more potent than SA. Contrary to HBE and Caco2, THP-1 are phagocytic cells and sub-micron to micron-sized agglomerates are more appropriate for phagocytosis by THP-1 than nano-sized agglomerates [28]. Therefore, we speculate that the increased uptake of LA via phagocytosis might account for these distinct responses. However, we did not notice such effects with 117 nm TiO_2_, possibly because the size of SA and LA was already optimal for phagocytosis (250 and 500 nm) and equally taken up by the cells. This indicates that the agglomeration state of TiO_2_ influences the biological responses not only depending on the cell type but also depending on the primary particle size. When summing up these in vitro results, we can conclude that, in any case, SA were not biologically more active than LA (Table 4A).

Studies reporting the influence of agglomeration on in vivo toxicity are scarce. Therefore, we exposed mice to differently agglomerated TiO_2_ NPs via two exposure routes and investigated acute toxic effects. Exposure to LA of 117 nm TiO_2_ via aspiration resulted in increased retention of Ti in the lung and a higher number of BAL lymphocytes compared to the SA. Inhalation studies recorded that large agglomerates of nano-TiO_2_ induced a stronger pulmonary response in rats than smaller ones [29, 30], which is in agreement with our findings. However, such differences were not observed for the 17 nm TiO_2_. Noël et al. [30] found that larger agglomerates of 10–30 nm and 50 nm sized TiO_2_ induced stronger pulmonary response than smaller and larger agglomerates of 5 nm sized TiO_2_ in a rat inhalation study. Thus, we can conclude that TiO_2_ agglomeration increases the pulmonary responses but depending on the primary particle size. In contrast, systemic DNA damage was influenced by the agglomeration state of both TiO_2_ but inversely. These results suggest that the influence of TiO_2_ agglomeration state on biological responses is not limited to the site of particle deposition, but could also affect systemic responses. Overall, SA did not induce more severe responses compared to LA except in one case (Table 4B).

Size has been identified as a major determinant of NM toxicity and their distribution is an important parameter to classify NMs according the EU definition [31]. To implement regulations and guidance in practice, standardization and validation of methods for measurement of the primary particle and AA characteristics, particularly their size, are essential to examine the effect of agglomeration on toxicity. However, not much attention has been paid to other size characterization, i.e. only reporting the least size detected but not the agglomeration status. DLS and PTA methods measure the hydrodynamic diameter of AA and estimate the AA size such as the diameter of sphere with equivalent hydrodynamic mobility. They do not measure the AA shape. As observed in our study, for particles that are agglomerated, DLS and PTA results are biased towards larger values [32]. The combination of TEM imaging and image analysis allows to more reliably analyse the size and shape of primary particles [33] and AA of TiO_2_ NPs [34]. Therefore, the TEM method was standardized and validated for the examined materials such that the suspensions examined in this study could be clearly differentiated, thus a reliable comparison of toxic/biological responses induced by differently sized agglomerates was possible.

In addition to the size differences between suspensions at the start of the exposure in vitro, evaluation of further agglomeration in cell exposure medium are crucial to determine their influence on toxicity [35–37]. In our study, we noticed significant agglomeration of both TiO_2_ only in Caco2 cell exposure medium. Cell exposure media with different compositions were shown to influence the stability of TiO_2_ NPs [38]. We suspect that the use of 1% non-essential amino acids (NEAA) in Caco2 cell exposure medium (necessary for the growth of Caco2 cells) might influence further agglomeration over time while such NEAA was absent in HBE or THP-1 cell exposure medium. When comparing epithelial cell types, i.e.HBE and Caco2, a slight difference in the magnitude of biological responses were observed, but further agglomeration did not lead to different effects as observed in THP-1. In vivo, it is more than likely that the size distribution of the gavaged suspensions is modified by passing through different regions of the digestive tract, where pH changes depending on the region. However, whether these in vivo modifications can be related to the change in in vitro size distribution in Caco-2 cell exposure medium can hardly be predicted.

Recent studies recognised effective density as a potential factor affecting the sedimentation and in vitro dosimetry (effective dose) [36, 39]. Therefore, we determined the effective density of TiO_2_ in all suspensions and simulated delivered in vitro doses using a distorted grid (DG) model [40]. The agglomeration state of both TiO_2_ had only a slight effect on the effective density of TiO_2_ and delivered doses. Further, the simulated delivered dose was not more than 60% of the nominal dose applied for all the TiO_2_ suspensions, indicating that the sedimentation/gravitational settling did not dominate the transport of the TiO_2_ NPs regardless of their agglomeration state. Based on these results, we can conclude that, the delivered in vitro doses were not a confounder in assessing the differential in vitro responses observed between LA and SA suspensions.

Each type of TiO_2_ NP is characterized by different physico-chemical properties and it exhibits different biological activities [41, 42]. Therefore, the results of this study may only be applicable to the TiO_2_ examined here. However, the approach and the dispersion methodology developed can be applied to investigate the (differential) toxicity of other types of NMs with regard to their agglomeration status.

## Conclusion

The dispersion protocol and descriptive TEM characterization used in this study allowed to investigate the influence of agglomeration state of TiO_2_ NPs on their toxicity/biological responses. According to the nanotoxicity paradigm, we hypothesized that small agglomerates would induce stronger toxicity than larger ones. Somewhat contra intuitively, we noted that, in most cases, no difference was found between agglomeration states and if any difference was found, large agglomerates mostly induced a stronger effect. In in vitro assays*,* the major differences were found in THP-1 cells, which is of interest in view of differential responses of innate immune cells. Also in in vivo assays*,* differential responses were noted both after respiratory and oral exposure. Thus, we conclude that agglomeration state of TiO_2_ NPs can influence their toxicity and that large agglomerates do not appear less active than small agglomerates. These results are, most probably, material and primary particle size specific, rather than agglomeration specific.

## Materials and methods

### TiO_2_ NPs

The European Commission’s Joint Research Centre (JRC, Italy) kindly provided the TiO_2_ representative test materials JRCNM10202a and JRCNM10200a. Mean primary particle sizes (Feret min) determined by TEM are 17 and 117 nm respectively and a detailed information on these particles is provided in the JRC report [43].

### Dispersion of NPs

A method based on a protocol developed by Guiot and Spalla [22] was used to generate two differently agglomerated suspensions of the same TiO_2_ NPs. Additional file 1: Figure S1 shows the changes in zeta potential of TiO_2_ NPs as a function of pH. The Zeta potential varies as a function of pH, which in turn determines the electrostatic interaction of particles with other particles and with the surrounding medium. Based on this principle, the agglomeration state over a range of pH (2–12) was analysed for each particle type using TEM, and pH conditions at which particles existed in different agglomeration states were selected.

Additional file 1: Figure S2 shows the schematic diagram of the modified Guiot and Spalla protocol to prepare our ad-hoc stock suspensions. After dispersing the particles in the respective pH solutions, the suspensions were sonicated, delivering 7056 J with a probe sonicator (Microson XL 2000, 3 mm probe, Belgium), and stabilized with 0.2 μm membrane filtered (Thermofischer Scientific, Belgium) 1% BSA (Sigma-Aldrich, Belgium). To ensure the delivery of the targeted energy and reproducibility of suspensions, probe sonicator was calorimetrically calibrated using the NANOREG protocol [44]. The experiments were repeated on different occasions and the variability of energy delivered between experiments was observed to be less than 10%. After sonication, the suspensions at pH 2 were readjusted to pH 7–7.5 by slowly adding sodium hydroxide solution (NaOH) 0.1 M. The stock suspensions were 2.56 mg TiO_2_/mL with 0.25% BSA and at pH 7–7.5, and freshly prepared for each independent experiment.

### Dynamic light scattering (DLS)

DLS measurements were performed with a ZetaSizer Nano ZS instrument (Malvern Instruments, Malvern, UK) to evaluate the size distribution of TiO_2_ NP in suspensions. Stock suspensions and TiO_2_ in cell culture medium (100 μg/mL) were tested for each condition. The settings were 2.4 for the refractive index and 0.2 for the absorption parameter. The selected dispersant was water (refractive index of 1.33). The mean hydrodynamic diameter (Z-average) and the polydispersity index (PDI) were measured with version 7.11 of the Zetasizer software.

### Transmission Electron microscopy (TEM)

TEM specimens of stock suspensions were prepared and examined using a well-aligned Tecnai Spirit microscope (FEI, Eindhoven, Netherlands) operating at 120 kV, at a spot size 3 and imaged in BF-mode in parallel beam conditions. Images were typically recorded at approximately 500 nm below minimal contrast conditions. Digital micrographs were made using the bottom-mounted 4 × 4 K Eagle CCD-camera and converted to tif-format using the TIA software. Equivalent circle (ECD) and Feret minimum diameter (Feret min) analyses were performed as described in [45].

### In vitro dosimetry

Volume Centrifugation Method (VCM) was used to determine the effective density of TiO_2_ NPs in cell culture medium (CCM) and the dose delivered to the cells over 24 h exposure was simulated using a Distorted Grid (DG) model, as described in [40].

### Cell culture conditions

The human bronchial epithelial cell line (16HBE14o- or HBE) and the human monocytic cell line (THP-1) were kindly provided by Dr. Gruenert (University of California, San Francisco, USA), and the Caucasian colon adenocarcinoma cell line (Caco2) (P.Nr: 86010202) was purchased from Sigma-Aldrich (Belgium). HBE cells were cultured in DMEM/F12 supplemented with 5% FBS, 1% penicillin-streptomycin (P-S) (100 U/mL), 1% L-glutamine (2 mM) and 1% fungizone (2.5 g/mL) while RPMI 1640 supplemented with 10% FBS, 1% P-S (100 U/mL), 1% L-glutamine (2 mM) and 1% fungizone (2.5 g/mL) was used for THP-1. DMEM/HG supplemented with 10% FBS, 1% P-S (100 U/mL), 1% L-glutamine (2 mM), 1% fungizone (2.5 g/mL) and 1% non-essential amino acids (NEAA) was used for Caco2 cells. All cell culture supplements were purchased from Invitrogen (Belgium) unless otherwise stated. Cells were cultured in T75 flasks (FALCON, USA) at 37 °C in a 100% humidified air containing 5% CO_2_. Medium was changed every 2 or 3 days and cells were passaged every week (7 days). Cells from passage 4 to 10 were used for the experiments.

### Exposure conditions in vitro

We used serum-free exposure media (culture medium without FBS) for in vitro conditions to avoid the influence of serum proteins on particle characteristics and biological responses. For all experiments except TEER measurement, HBE, Caco2 and THP-1 cells were seeded at a density of 1.5 × 10^5^, 1.05 × 10^5^ cm^2^ (surface area of culture well) and 3.3 × 10^5^/mL in 96 or 24 well plates (greiner bio-one, Belgium) and incubated overnight. On the day of exposure, freshly prepared stock suspensions were diluted in BSA 0.25% to prepare the sub-stocks of different concentrations (40 to 2560 μg/mL) and further diluted 10 times in serum-free exposure media to achieve the final exposure concentrations (4 to 256 μg/mL). BSA 0.25% diluted 10 times in exposure media served as negative control. Cells were then washed with HBSS (without Ca^2+^/Mg^2+^) once and exposed to TiO_2_ NPs. After 24 h, cell cultures were washed twice with HBSS and the respective assays were performed.

### Animals and treatments

Female C57BL/6JRj mice were purchased from Janvier Labs (St Bertevin, France). Eight-week-old animals were kept with sterile rodent feed and acidified water, and housed in positive-pressure air-conditioned units (25 °C, 50% relative humidity) on a 12 h light/dark cycle. For aspiration, mice were anaesthesized with a mix of Nimatek, 1 mg/mouse (Eurovet, Bladel, Netherlands) and Rompun, 0.2 mg/mouse (Bayer, Kiel, Germany) given intraperitoneally, and administered a 50 μl suspension of particles or suspension medium at pH 7.5 (controls) by oro-pharyngeal aspiration. For gavage, mice were administered 200 μL suspension or dispersion medium. Mice were sacrificed 3 d after particle administration with an intraperitoneal injection of 12 mg sodium pentobarbital (Certa, Braine-l’Alleud, Belgium).

### Blood, broncho-alveolar lavage and organ sampling

Blood was collected in EDTA tubes for inductively coupled plasma-mass spectrometry (ICP-MS), hematology and genotoxicity (comet assay). Broncho-alveolar lavage (BAL) was performed in mice treated by oro-pharyngeal aspiration cannulating the trachea and infusing the lungs with 1 ml NaCl 0.9%. Whole lungs were then perfused with NaCl 0.9% and excised. Left lobes were placed in 3.65% paraformaldehyde (Sigma-Aldrich, St Louis, Missouri, USA) in phosphate buffered saline (PBS) for later histological analysis, and right lobes were used for ICP-MS, and total glutathione (GSH) measurements. BAL were centrifuged 10 min at 4 °C (240 *g*). Cell-free supernatant (BALF) was used for biochemical measurements including lactate dehydrogenase (LDH) activity and total proteins (Cobas 8000, Roche Diagnostics). After resuspension in PBS, total BAL cells were counted in Turch (crystal violet 1%, acetic acid 3%) and cytocentrifuged for differentiation by light microscopy after Diff-Quick staining (200 cells counted, Polysciences, Warrington, UK). Liver, spleen and kidney were collected after gavage for ICP-MS and GSH measurement (liver).

### Inductively coupled plasma mass-spectrometry (ICP-MS)

200 mg mouse lung tissue were mineralized with 4.5 ml HNO_3_ 65% and 1.5 ml HCl 30% in a microwave (Multiwave Go, Anton Paar) before ICP-MS measurement. Ti recovery and the effect of the biological matrix were determined in preliminary experiments (data not shown). Ti was quantified on an ICP-MS Agilent 7500 ce Octopole Reaction System according to the following method: method spectrum, analyte 47Ti, internal standard 74Ge, Helium mode, peak pattern maximum (20), integration time 1 s per point/20 s per mass, acquisition time: 5 repetitions. Calibration of the measurement was done with serial dilutions of a TraceCERT Titanium Standard for ICP (soluble Ti).

### Cell metabolic activity

To determine cell metabolic activity, supernatants were removed after 24 h exposure and cells were incubated with 120 μL water soluble tetrazolium salts (WST-1) reagent (Roche, Belgium) diluted in medium without phenol red at the ratio of 1:10. After 1 to 2 h incubation, plates were centrifuged at 1600 *g* for 10 min, 100 μL was transferred to a new plate and the optical density (OD) was recorded using a micro-plate reader (Bio-Rad, USA) at 450 nm. After subtracting the blank OD values from the sample OD values, results were expressed as percentage of control (untreated) cells.

### Effect on cell viability

Cell viability was assessed by cellular leakage of LDH using a kinetic assay [46]. At the end of exposure, supernatants were transferred to a new plate and cells were incubated with triton 0.2% (Sigma-Aldrich, Belgium). After 30 min, plates were centrifuged at 1600 g for 10 min. After transfer to a new plate, freshly prepared substrate solution (pyruvate+NADH) was added and the absorbance was measured by a spectrophotometer at 340 nm for 3 min with 15 s interval. Slope was calculated according to the standard curve. Cell viability was calculated as

$$ {\displaystyle \begin{array}{c}\left[\mathrm{slope}\ \mathrm{of}\ \mathrm{leakage}/\left(\mathrm{slope}\ \mathrm{of}\ \mathrm{lysate}+\mathrm{slope}\ \mathrm{of}\ \mathrm{leakage}\right)\ast 100\right]\\ {}\mathrm{and}\ \mathrm{relative}\ \mathrm{viability}\ \mathrm{as}\left(\mathrm{sample}\ \mathrm{viability}/\mathrm{untreated}\ \mathrm{control}\ \mathrm{viability}\right)\ast 100\end{array}} $$

### Total glutathione measurements

Total glutathione (GSH) is a cellular antioxidant, which is depleted when excessive reactive oxygen species (ROS) are produced. Therefore, GSH depletion was measured as an indicator of oxidative stress induction [47] using a GSH detection kit (Enzo life sciences, Belgium). After 24 h exposure, cell cultures were washed and harvested using trypsin 0.1% (Gibco, Belgium). For in vivo, a part of the lung and liver was sliced and weighted. Then, cells/tissues were resuspended in metaphosphoric acid 5% and homogenized using an ultra turrax t25 tissue homogenizer (Janke & kunkel, Germany). GSH was quantified according to manufacturer’s protocol and the protein content of cell cultures was assessed using bicinchoninic acid (BCA) protein assay kit (Thermo Fischer Scientific, Pierce, Belgium). GSH was normalized to the total protein content in vitro and the results were expressed as percentage of control (untreated) cells. For in vivo*,* GSH was normalized to the mass of lung or liver tissue. Cells treated with Tert-Butylhydroquinone (t-BHQ) 100 μM for 24 h were used as positive control (data not shown).

### Cytokine quantification

As indicators of pro-inflammatory responses, interleukins (IL) -8, IL-6, IL-1β and tumor necrosis factor-alpha (TNF-α) levels were measured using enzyme-linked immunosorbent assay (ELISA) kits (Sigma-Aldrich, Belgium) in cell supernatants according to manufacturer’s protocol. Results were normalized to the total protein content and expressed as a ratio to control (untreated) cells. Cells treated with LPS 1 μg/mL for 24 h were used as positive control (data not shown).

### Trans-epithelial electrical resistance

Trans-epithelial electrical resistance (TEER) was measured in epithelial (HBE and Caco2) monolayers as an estimation of epithelial barrier integrity. HBE and Caco2 cells were seeded at a density of 2 × 10^4^ cells per well in 24 well transwell inserts (0.4 μm pore size, polyester membrane, Corning, CLS3470 Sigma). TEER was monitored everyday using a Chopstick electrode and an epithelial voltohmmeter (EVOM) (World Precision Instruments, Sarasota, USA). After 7 days, cultures with TEER > 600 Ω.cm^2^ were exposed to different concentrations of TiO_2_ suspensions for 24 h and TEER was measured. Cultures exposed to sodium dodecyl sulfate (SDS) 200 μg/mL for 24 h served as positive control for epithelial barrier disruption (data not shown). Results are expressed as percentage of control (untreated) cells.

### Comet assay

In earlier studies, cellular and in some cases nuclear uptake of TiO_2_ has been shown [48–51]. In our study, we verified cellular internalization in HBE cells (Additional file 1: Figure S9). DNA strand breaks were quantified as a measure of DNA damage. Cell cultures exposed to non-cytotoxic NM concentrations (5, 25, 50 and 100 μg/mL) were used to quantify DNA strand breaks using alkaline comet assay kit (Trevigen, C.No.4250–050-K) according to manufacturer’s protocol. Cells treated with methyl methane sulfonate (MMS, Sigma-Aldrich, Belgium) 100 μM for 1–2 h served as positive control. For in vivo experiments*,* comet assay was performed on blood and BAL cells collected from animals. Untreated animal blood or BAL cells exposed to H_2_O_2_ 100 μM for 15 min served as positive control. Slides were imaged using microscopy (BX61, Olympus, Belgium) in FITC mode and at 10x magnification. Casplab software version casplab_1.2.3beta2 (http://casplab.com/download) was used to score 50 comets per well. The mean percentage of tail DNA was calculated from the median of three independent experiments.

### Statistical analysis

For in vitro assays, three independent experiments were performed in triplicate or duplicate and data was presented as mean ± standard deviation (SD). For in vivo, mean ± SD was calculated for 4–5 animals per group. Using GraphPad prism 7 software (https://www.graphpad.com/), results were analysed with one-way ANOVA followed by a Dunnett’s multiple comparison test to determine the significance of differences compared with control. Two-way ANOVA followed by Bonferroni’s multiple comparison test was used to determine significance of differences between suspensions (see Table 4 for explanation).

## Supplementary information

**Additional file 1 **: **Table S1.** Main parameters necessary to calculate the delivered dose in vitro for different the TiO_2_ suspensions. **Figure S1.** pH vs Zeta potential curves. 17 nm TiO_2_ (A) and 117 nm TiO_2_ (B). **Figure S2.** Scheme of the protocol for the preparation of SA and LA from TiO_2_ suspensions. To obtain small (SA) and large agglomerates (LA), 17 and 117 nm TiO_2_ were dispersed at different pH conditions, sonicated and stabilized with BSA 0.25%. The suspensions dispersed at pH 2 were readjusted to pH 7–7.5 using 0.1 M NaOH. **Figure S3.** Influence of TiO_2_ agglomeration on cytotoxicity in vitro. WST-1 and LDH assay were used to measure the cell metabolic activity in HBE (A), Caco2 (C) and THP-1 (E) and cell viability in HBE (B) Caco2 (D) and THP1 (F) after 24 h exposure to small (SA) and large agglomerates (LA) of 17 nm and 117 nm TiO_2_. Data are expressed as means ± SD from three independent experiments performed in triplicates. *p* < 0.001 (***) represents significant difference compared to control (One-way ANOVA followed by Dunnett’s multiple comparison test). **Figure S4.** Influence of TiO_2_ agglomeration on total glutathione (GSH) in vitro. GSH depletion was measured as an indicator of oxidative stress in HBE (A,B) and Caco2 (C) cells after 24 h exposure to small (SA) and large agglomerates (LA) of 17 nm (A,C) or 117 nm TiO_2_ (B). Data are expressed as means ± SD from three independent experiments performed in duplicates. *p* < 0.05 (*), *p* < 0.01 (**) and *p* < 0.001 (***) represent significant difference compared to control (One-way ANOVA followed by Dunnett’s multiple comparison test). **Figure S5.** Influence of TiO_2_ agglomeration on barrier integrity in epithelial monolayers in vitro. Trans-epithelial electrical resistance (TEER) was measured in HBE (A, B) and Caco2 (C) after 24 h exposure to small (SA) and large agglomerates (LA) of 17 nm (A) or 117 nm TiO_2_ (B, C). Data are expressed as means ± SD from three independent experiments performed in duplicates. *p* < 0.05 (*), *p* < 0.01 (**) and *p* < 0.001 (***) represent significant difference compared to control (One-way ANOVA followed by Dunnett’s multiple comparison test). **Figure S6.** Influence of TiO_2_ agglomeration on cytokine release in vitro. TNF-α (A), IL-6 (B) and IL-β (C) levels were measured in the supernatant of the HBE (A,B) and THP-1 (C) after 24 h exposure to small (SA) and large agglomerates (LA) of 17 nm (A, C) or 117 nm TiO_2_ (B). Data are expressed as means ± SD from three independent experiments performed in duplicates. *p* < 0.05 (*), *p* < 0.01 (**) and *p* < 0.001 (***) represent significant difference compared to control (One-way ANOVA followed by Dunnett’s multiple comparison test). **Figure S7.** Influence of TiO_2_ agglomeration on DNA damage in vitro. DNA damage was measured in HBE (A,C), Caco2 (B,D) and THP-1 (E) after 24 h exposure to small (SA) and large agglomerates (LA) of 17 nm (A,B) or 117 nm TiO_2_ (C, D,E). Data are expressed as means ± SD from three independent experiments performed in duplicates. *P* < 0.05 (*), *p* < 0.01 (**) and *p* < 0.001 (***) represent significant difference compared to control (One-way ANOVA followed by Dunnett’s multiple comparison test). **Figure S8.** Influence of TiO_2_ agglomeration on in vivo toxicity in mice exposed via oropharyngeal aspiration. BAL lymphocytes (A), Ti persistence in lung tissues (B) and BALF LDH activity (C) measured after 3 d in mice aspirated with different doses of small (SA) and large agglomerates (LA). Data are expressed as means ± SD from 4 to 5 mice in each group. *p* < 0.05 (*), *p* < 0.01 (**) and *p* < 0.001 (***) represent significant difference compared to control (One-way ANOVA followed by Dunnett’s multiple comparison test). **Figure S9.** Intracellular uptake of TiO_2_ agglomerates by HBE cell cultures and cellular distribution. TEM images of control cells (A) and exposed to 50 μg/mL of TiO_2_ NPs for 24 h: 17 nm-SA (B), 17 nm-LA (C), 117 nm-SA (D) and 117 nm-LA (E). N -Nucleus; C-Cytoplasm. Some TiO_2_ agglomerates close to the nucleus induced arch like structures (indicated in red arrow).

## Data Availability

The datasets used and/or analysed during the current study are available from the corresponding author on reasonable request.

## Abbreviations

AA: Agglomerates and aggregates
ANOVA: Analysis of variance
BAL: Broncho-alveolar lavage
BALF: BAL fluid
BCA: Bicinchoninic acid, assay
BSA: Bovine serum albumin
CCD: Charge coupled device
CCM: Cell culture medium
DG: Distorted grid
DLS: Dynamic light scattering
ECD: Equivalent circle diameter
EDTA: Ethylenediaminetetraacetic acid
ELISA: Enzyme linked immunosorbent assay
FBS: Fetal bovine serum
Feret min: Minimum feret diameter
GSH: Total glutathione
HBSS: Hank’s balanced salt solution
IARC: International agency for research on cancer
ICP-MS: Inductively coupled plasma-mass spectrometry
IL: Interleukin
JRC: Joint research commission
LA: Large agglomerates
LDH: Lactate dehydrogenase
LPS: Lipopolysaccharide
MMS: Methylmethane sulfonate
NMs: Nanomaterials
NPs: Nanoparticles
OD: Optical density
PBS: Phosphate buffer saline
PDI: Polydispersity index
P-S: Penicillin-streptomycin
PTA: Particle tracking analysis
ROS: Reactive oxygen species
SA: Small agglomerates
SD: Standard deviation
TEER: Transepithelial electrical resistance
TEM: Transmission electron microscopy
TNF: Tumor necrosis factor
VCM: Volume centrifugation method
WST-1: Water soluble tetrazolium salts 1
Z-average: Mean hydrodynamic diameter
ZS: Zeta sizer
