# Supplementary material for: Agglomeration of titanium dioxide nanoparticles increases toxicological responses in vitro and in vivo
Source: Part Fibre Toxicol. 2020 Feb 26;17:10. doi: 10.1186/s12989-020-00341-7 (PMC7045370; doi:10.1186/s12989-020-00341-7)
Supplement: Supplementary file 1 — Additional file 1 : Table S1. Main parameters necessary to calculate the delivered dose in vitro for different the TiO2 suspensions. Figure S1. pH vs Zeta potential curves. 17 nm TiO2 (A) and 117 nm TiO2 (B). Figure S2. Scheme of the protocol for the preparation of SA and LA from TiO2 suspensions. To obtain small (SA) and large agglomerates (LA), 17 and 117 nm TiO2 were dispersed at different pH conditions, sonicated and stabilized with BSA 0.25%. The suspensions dispersed at pH 2 were readjusted to pH 7–7.5 using 0.1 M NaOH. Figure S3. Influence of TiO2 agglomeration on cytotoxicity in vitro. WST-1 and LDH assay were used to measure the cell metabolic activity in HBE (A), Caco2 (C) and THP-1 (E) and cell viability in HBE (B) Caco2 (D) and THP1 (F) after 24 h exposure to small (SA) and large agglomerates (LA) of 17 nm and 117 nm TiO2. Data are expressed as means ± SD from three independent experiments performed in triplicates. p < 0.001 (***) represents significant difference compared to control (One-way ANOVA followed by Dunnett’s multiple comparison test). Figure S4. Influence of TiO2 agglomeration on total glutathione (GSH) in vitro. GSH depletion was measured as an indicator of oxidative stress in HBE (A,B) and Caco2 (C) cells after 24 h exposure to small (SA) and large agglomerates (LA) of 17 nm (A,C) or 117 nm TiO2 (B). Data are expressed as means ± SD from three independent experiments performed in duplicates. p < 0.05 (*), p < 0.01 (**) and p < 0.001 (***) represent significant difference compared to control (One-way ANOVA followed by Dunnett’s multiple comparison test). Figure S5. Influence of TiO2 agglomeration on barrier integrity in epithelial monolayers in vitro. Trans-epithelial electrical resistance (TEER) was measured in HBE (A, B) and Caco2 (C) after 24 h exposure to small (SA) and large agglomerates (LA) of 17 nm (A) or 117 nm TiO2 (B, C). Data are expressed as means ± SD from three independent experiments performed in duplicates. p < 0.05 (*), p < [file 12989_2020_341_MOESM1_ESM.docx]

**ADDITIONAL FILE 1:**

**AGGLOMERATION OF TITANIUM DIOXIDE NANOPARTICLES INCREASES TOXICOLOGICAL RESPONSES *IN VITRO* AND *IN VIVO***

Sivakumar Murugadoss^1^, Frederic Brassinne^2^, Noham Sebaihi^3^, Jasmine Petry^3^, Stevan M. Cokic^4^ , Kirsten L. Van Landuyt^4^, Lode Godderis^5,7^, Jan Mast^2^, Dominique Lison^6^, Peter H. Hoet^1,§^ , Sybille van den Brule^6,§^

^1^Laboratory of Toxicology, Unit of Environment and Health, Department of Public Health and Primary Care, KU Leuven, 3000 Leuven, Belgium

^2^Trace Elements and Nanomaterials, Sciensano, 1180 Uccle, Belgium

^3^National Standards, FPS Economy, 1000 Brussels, Belgium

^4^KU Leuven, Department of Oral Health Sciences, BIOMAT & UZ Leuven (University Hospitals Leuven), Dentistry, Kapucijnenvoer 7, 3000 Leuven, Belgium

^5^Laboratory for Occupational and Environmental Hygiene, Unit of Environment and Health, Department of Public Health and Primary Care, KU Leuven, 3000 Leuven, Belgium

^6^Louvain centre for Toxicology and Applied Pharmacology, Institute of Experimental and Clinical Research, Université catholique de Louvain, 1200 Brussels, Belgium

^7^IDEWE, External Service for Prevention and Protection at work, Interleuvenlaan 58, 3001 Heverlee, Belgium

**Materials and Methods:**

### TiO_2_ uptake studies:

HBE cells exposed to 50 µg/mL of TiO_2_ suspensions for 24 h were fixed with 2.5% formaldehyde in DPBS buffer (without Ca^2+^/Mg^2+^). Samples were washed three times using the same buffer, fixed in 1% OsO4 and dehydrated in ascending concentrations of ethanol. Subsequently, the cells were embedded in epoxy resin (Sigma Aldrich Chemie Gmbh, Steinheim, Germany). Sections of 50-70 nm were obtained by ultramicrotomy using a diamond knife and images were acquired using TEM (JEOL, JEM-1200 EX II Tokyo, Japan) at a voltage of 80 kV.

**Table S1: Main parameters necessary to calculate the delivered dose *in vitro* for different the TiO_2_ suspensions.**

|  | DMEM/F12 | | | | RPMI 1640 | | | |
| --- | --- | --- | --- | --- | --- | --- | --- | --- |
| Suspension | 17nm  SA | 17nm  LA | 117nm  SA | 117nm  LA | 17nm  SA | 17nm  LA | 117nm  SA | 117nm  LA |
| Effective density (g/cm^3^) | 1.5519 | 1.4939 | 1.7814 | 1.6804 | 1.4896 | 1.4634 | 1.6655 | 1.6543 |
| Medium density (g/cm^3^) | 1.0211 | 1.0211 | 1.0211 | 1.0211 | 1.0102 | 1.0102 | 1.0102 | 1.0102 |
| Medium viscosity (mPa S) | 0.93 | 0.93 | 0.93 | 0.93 | 0.95 | 0.95 | 0.95 | 0.95 |

Effective density of TiO_2_ in exposure media and, density and viscosity of exposure media prepared from DMEM/F12 and RPMI 1640 were calculated. All dosimetry simulations were performed for 24 h incubation at 37°C and for a 6 mm liquid column height. The hydrodynamic sizes are given in table 2.

**Figure S1: pH vs Zeta potential curves.** 17 nm TiO_2_ (A) and 117 nm TiO_2_ (B).

**Figure S2: Scheme of the protocol for the preparation of SA and LA from TiO_2_ suspensions.** To obtain small (SA) and large agglomerates (LA), 17 and 117nm TiO_2_ were dispersed at different pH conditions, sonicated and stabilized with BSA 0.25%. The suspensions dispersed at pH 2 were readjusted to pH 7-7.5 using 0.1M NaOH.

**Figure S3: Influence of TiO_2_ agglomeration on cytotoxicity *in vitro*.** WST-1 and LDH assay were used to measure the cell metabolic activity in HBE (A), Caco2 (C) and THP-1 (E) and cell viability in HBE (B) Caco2 (D) and THP1 (F) after 24 h exposure to small (SA) and large agglomerates (LA) of 17 nm and 117nm TiO_2_. Data are expressed as means ± SD from three independent experiments performed in triplicates. p < 0.001 (***) represents significant difference compared to control (One-way ANOVA followed by Dunnett’s multiple comparison test).

**Figure S4: Influence of TiO_2_ agglomeration on total glutathione (GSH) *in vitro*.** GSH depletion was measured as an indicator of oxidative stress in HBE (A,B) and Caco2 (C) cells after 24 h exposure to small (SA) and large agglomerates (LA) of 17 nm (A,C) or 117 nm TiO_2_ (B). Data are expressed as means ± SD from three independent experiments performed in duplicates. p < 0.05 (*), p < 0.01 (**) and p < 0.001 (***) represent significant difference compared to control (One-way ANOVA followed by Dunnett’s multiple comparison test).

**Figure S5: Influence of TiO_2_ agglomeration on barrier integrity in epithelial monolayers *in vitro*.** Trans-epithelial electrical resistance (TEER) was measured in HBE (A, B) and Caco2 (C) after 24 h exposure to small (SA) and large agglomerates (LA) of 17 nm (A) or 117nm TiO_2_ (B, C). Data are expressed as means ± SD from three independent experiments performed in duplicates. p < 0.05 (*), p < 0.01 (**) and p < 0.001 (***) represent significant difference compared to control (One-way ANOVA followed by Dunnett’s multiple comparison test).

**Figure S6: Influence of TiO_2_ agglomeration on cytokine release *in vitro*.** TNF-α (A), IL-6 (B) and IL-β (C) levels were measured in the supernatant of the HBE (A,B) and THP-1 (C) after 24 h exposure to small (SA) and large agglomerates (LA) of 17 nm (A, C) or 117nm TiO_2_ (B). Data are expressed as means ± SD from three independent experiments performed in duplicates. p < 0.05 (*), p < 0.01 (**) and p < 0.001 (***) represent significant difference compared to control (One-way ANOVA followed by Dunnett’s multiple comparison test).

**Figure S7: Influence of TiO_2_ agglomeration on DNA damage in vitro.** DNA damage was measured in HBE (A,C), Caco2 (B,D) and THP-1 (E) after 24 h exposure to small (SA) and large agglomerates (LA) of 17 nm (A,B) or 117nm TiO_2_ (C, D,E). Data are expressed as means ± SD from three independent experiments performed in duplicates. P < 0.05 (*), p < 0.01 (**) and p < 0.001 (***) represent significant difference compared to control (One-way ANOVA followed by Dunnett’s multiple comparison test).

**Figure S8: Influence of TiO_2_ agglomeration on in vivo toxicity in mice exposed via oropharyngeal aspiration.** BAL lymphocytes (A), Ti persistence in lung tissues (B) and BALF LDH activity (C) measured after 3 d in mice aspirated with different doses of small (SA) and large agglomerates (LA). Data are expressed as means ± SD from 4-5 mice in each group. p < 0.05 (*), p < 0.01 (**) and p < 0.001 (***) represent significant difference compared to control (One-way ANOVA followed by Dunnett’s multiple comparison test).

**Figure S9: Intracellular uptake of TiO_2_ agglomerates by HBE cell cultures and cellular distribution.** TEM images of control cells (A) and exposed to 50 μg/mL of TiO_2_ NPs for 24 h: 17nm-SA (B), 17nm-LA (C), 117nm-SA (D) and 117nm-LA (E). N -Nucleus; C-Cytoplasm. Some TiO_2_ agglomerates close to the nucleus induced arch like structures (indicated in red arrow).
